# Supplementary material for: A critical role of FAK signaling in Rac1-driven melanoma cell resistance to MAPK pathway inhibition
Source: Oncogene. 2025 Oct 18;44(46):4520–32. doi: 10.1038/s41388-025-03603-w (PMC12602353; doi:10.1038/s41388-025-03603-w)
Supplement: Supplementary file 1 — Supplemental Figures and Legends [file 41388_2025_3603_MOESM1_ESM.pdf]

Supplementary Data

Figure S1. The PAK1-specific inhibitor G-5555 does not block the growth of Vav1-overexpressing cells on its own. Vector control (**VEC**), Vav1 overexpressing cells (**VAV1**), or Vav1 overexpressing A375 cells were treated with DMSO vehicle or 1 μM G-5555. Fold change in tumor cell number was measured on day 9 via resazurin assay.

Figure S2. Rac1 P29S-driven BRAFi resistance in 451-Lu cells is partially PAK1-dependent. (A) 451-Lu vector control cells (**VEC**), or cells harboring Rac1 P29S (**P29S**) were treated with 5 μM vemurafenib (**VEM**) or 5 μM vemurafenib plus 1 μM G-5555 (**VEM + G-5555**). Fold change in tumor cell number was measured on day 6 via resazurin assay. Treatment with vemurafenib plus G-5555 reduced cell proliferation compared to treatment with vemurafenib alone; *P<0.05, ****P<0.0001, ANOVA with Sidak's multiple comparison test. **(B)** The same cell lines as in (A) were treated with DMSO vehicle control or 1 μM G-5555 alone. **(C)** Lysates of vector control and Rac1 P29S cells were analyzed by SDS-PAGE and immunoblotting for phospho-MEK S298, total MEK1, and tubulin loading control. Rac1 P29S increased phosphorylation at PAK-controlled MEK S298, and this was largely abolished in cells treated with G-5555.

Figure S3. A MEK BRAF phosphomimetic, but not a MEK PAK kinase phosphomimetic is sufficient to drive BRAFi resistance in A375 cells. A population doubling versus time experiment showed that enforced expression of a MEK1 S217D/S221D mutant, a phosphomimetic at the BRAF-controlled serine-217 and 221 phosphorylation sites, promoted rapid proliferation in the presence of 3 μM vemurafenib. In contrast, enforced expression of a MEK1 S298D mutant, intended to mimic phosphorylation of MEK1 at the PAK kinase-controlled serine-298 phosphorylation site, failed to promote proliferation in the presence of 3 μM vemurafenib any better than enforced expression of wild type MEK1. See Table S2 for area under the curve (AUC) statistical analysis of differences in growth curves.

Figure S4. Lack of effect of MEK knockdown on VAV1-driven BRAFi resistance. (**A**) The graph shows a population doubling versus time experiment using scaled up cultures of A375 vector control cells (**VEC**), Vav1-overexpressing cells (**VAV1**), and Vav1-overexpressing cells with RNAi knockdown of MEK1 (**MEK1 KD**) or both MEK1 and MEK2 (**MEK1/2 DKD**) (one large flask per cell type, growing in 3 μM vemurafenib with 3 nM cobimetinib). VAV1 cells doubled

nearly 6 times by day 21, while vector control cells doubled less than twice. The MEK knockdown cell lines doubled 8 times. (**B**) Lysates prepared from the cell lines in panel A growing in vehicle control (**DMSO**) or 3 μM vemurafenib with 3 nM cobimetinib (**Vem/Cobi**) overnight or for 21 days (assay endpoint) were analyzed by SDS-PAGE followed by immunoblotting for the indicated proteins. VAV1 overexpression increased levels of phospho-ERK and phospho-MEK in vem/cobi-treated cells, and this was reversed by MEK knockdown.

Figure S5. Characteristics of spontaneously BRAFi-resistant A375 sublines. (A) Lysates of parental A375 cells (A375) and A375 cells that acquired spontaneous BRAFi resistance by a variety of different mechanisms were analyzed by SDS-PAGE and immunoblotting. Spontaneous resistance mechanisms identified included tandem duplication of the BRAF kinase domain (BRAF Tdup) in EC1.1, BRAF N-terminal truncation (ΔN-BRAF) in EC2.1, loss of RasGAP NF1 in ES2.2.1 and ES2.2.2 and increased signaling towards the PAK-controlled pMEK S298 site in the absence of the other mechanisms (VRPP1-3). Cells were either untreated, or treated with 3 μM vemurafenib, as indicated. **(B)** Gene expression was analyzed via RNA seq for parental A375 cells treated or not with 3 μM vemurafenib compared to each of the BRAFi resistant populations maintained under vemurafenib treatment. Unsupervised hierarchical clustering was performed based on levels of the top 100 variably expressed genes across all samples. The analysis revealed that the VRPP sublines display a distinct gene expression profile, characterized in part by increased expression of TEAD1/AP1 target genes. Statistical significance of this YAP/TAZ pathway enrichment was assessed by gene set enrichment analysis (GSEA) **(C)** As described in the text, the VRPP3 subline was found to have acquired a Rac1 N92I mutation. Knockdown of Rac1 in VRPP3 via three different retrovirally delivered shRNAs strongly inhibited growth under both control (DMSO) conditions and in the presence of 3 μM vemurafenib (VEM). Inset shows strong knockdown of Rac1 in each of the sublines. **(D)** VRPP2 or VRPP3 cells were treated with DMSO vehicle, 3 μM vemurafenib (vem), 3 μM vemurafenib plus 3 nM cobimetinib (vem/cobi), or 3 μM vemurafenib plus 2 μM saracatinib (vem/sara). Fold change in cell number was measured on day 7 via resazurin assay. Both cell lines were resistant to vemurafenib and cobimetinib, but both were strongly blocked by the combination of vemurafenib and saracatinib; ***$P<0.001$, ****$P<0.0001$, ANOVA with Sidak's multiple comparison test.

Figure S6. VRPP subline gene expression indicates transition from neural crest-like to undifferentiated state. (A) Unsupervised hierarchical clustering was performed on RNA seq

data from parental A375 cells and BRAFi resistant sublines based on gene sets identified by Tsoi et al. to represent four distinct stages of melanoma cell differentiation. Across these gene sets, VRPP sublines 1-3 clustered together. **(B)** Gene set enrichment analysis (GSEA) revealed strong, statistically significant enrichment for higher expression of “undifferentiated” genes and lower expression of “neural crest-like” genes in VRPP lines, as compared to all other samples.

Figure S7. Ulixertinib inhibition of p90RSK phosphorylation in BRAFi-resistant A375 cell lines. Vector control A375 cells, cells expressing Vav1 or Rac1 P29S, and the spontaneously BRAFi-resistant sublines VRPP3 and EC2.1 were treated with DMSO or 1, 3, or 5 µM ulixertinib overnight and then lysates were analyzed by immunoblotting for phospho-P90 RSK, total p90 RSK1, or tubulin. Ulixertinib treatment suppressed phospho-P90RSK to a similar extent in all five cell lines, as observed by blotting for phospho-S380 directly, but also by the electrophoretic shift in total p90RSK1. Interestingly, EC2.1, which harbors a BRAF truncation mutant, had by far the highest level of phospho-S380, but was also the most sensitive to ulixertinib, as shown in Figure 6.

Figure S8. Rac1 P29S promotes resistance to ERK inhibition in 451-Lu cells. 451-Lu vector control (VEC) and Rac1 P29S cells were treated with 1, 3, or 5 µM ulixertinib, and fold change in cell growth was measured over time via resazurin assay. Compared to control cells, Rac1 P29S expressing cells were partially resistant to 1 µM ulixertinib treatment, as analyzed on day 11, ***P=0.0003, unpaired t-test.

Figure S9. BRAFi resistance of A375 VRPP3 cells is partially dependent on YAP and TAZ expression. (A) VRPP3 cells expressing a non-targeting shRNA (NT), or three different shRNAs each targeting YAP or TAZ were treated with 3 µM vemurafenib. Fold change in cell number was measured on day 6 via resazurin assay. All 6 shRNAs targeting YAP or TAZ partially suppressed the growth of the cells in vemurafenib; ****P<0.0001, ANOVA with Sidak’s multiple comparison test. (**B**) Analysis of lysates of NT control cells and YAP or TAZ knockdown cells vis SDS-PAGE and immunoblotting showed effective knockdown of YAP and TAZ in cells with the YAP or TAZ shRNAs. In the TAZ knockdown cells, YAP was significantly upregulated compared to the level observed in the NT control cells. A tubulin loading control confirmed equal loading in each lane. (**C**) Control cells and LATS-deleted cells (ΔLATS) were treated with DMSO, 1 µM ulixertinib, or 3 µM ulixertinib, and lysates were blotted for the indicated proteins. (**D**) In a growth assay of control cells and ΔLATS cells treated with DMSO or 3 µM vemurafenib

(VEM), the ∆LATS cells showed substantial resistance to vemurafenib compared to vector control cells as analyzed on day 10, ****$P<0.0001$, unpaired t test. (**E**) The ∆LATS cells also displayed substantial resistance to ulixertinib treatment, as analyzed in a separate assay. (Note this assay was performed together with the assays shown in Figure 6, sharing the vector controls).

Figure S10. Analysis of different MAP kinase signaling pathways in A375 EC2.1 and VRPP3 cells. Lysates of A375 parental cells, EC2.1 cells with a BRAF truncation, and VRPP3 cells with a Rac1 N92I mutation were analyzed by SDS-PAGE and immunoblotting. Cells were untreated or treated with 3 μM vemurafenib for 2 or 6 days. Phospho-p90 RSK S380, an ERK1/2 target, was maintained in the EC2.1 cells, but not strongly expressed in VRPP3 cells regardless of drug treatment. In contrast, phospho-JUN and phospho-P38 MAP kinase appeared increased in VRPP3 cells. Phospho-MEK S217, a BRAF-controlled site, was strongly maintained in EC2.1 cells, but reduced VRPP3 cells. Both phospho- and total ERK1 appeared somewhat reduced in VRPP3 compared to the other sublines. Overall, the differences in MAP kinase signaling in VRPP3 appeared consistent with a reduced reliance on ERK signaling in those cells.

Figure S11. The A375 VRPP3 subline utilizes multiple MAP kinase pathways to resist drug treatments. (A) Parental A375 or the VRPP3 subline were treated with DMSO vehicle, or different combinations of BRAF inhibitor vemurafenib (3 μM; VEM), MEK inhibitor cobimetinib (3 nM: COBI), Jun kinase inhibitor JNK-IN-8 (3 μM) or p38 MAP kinase inhibitor LY2228820 (5 μM; LY). **(B)** A separate experiment from that shown in (A), using an additional treatment in which BRAF, p38 MAP kinase, and Jun kinase were all inhibited simultaneously. Only this triple combination was able to completely suppress the drug resistant phenotype of the VRPP3 cells.

Figure S12. Inhibition of FAK can block the BRAFi resistance of Rac1-driven melanoma cells. (**A-G)** A375 parental cells (A), VRPP1-3 sublines (B-D), A375 Vav1 overexpressing cells (E), or 451-Lu vector or Rac1 P29S cells (F-G) were treated with DMSO vehicle (CON), 3 μM vemurafenib (A375 cells) or 5 μM vemurafenib (451-Lu cells) (VEM), 2 μM defactinib (DEF 2.0), 4 μM defactinib (DEF 4.0), or vemurafenib combined with defactinib (VEM/DEF 2.0 and VEM DEF 4.0). Fold change in cell growth was measured over time via resazurin assay. Co-treatment with defactinib blocked resistance to vemurafenib and in some cases cells responded to defactinib treatment alone. **(H&I)** In a similar experiment, defactinib was replaced with the FAK-

specific inhibitor PF 573228 at 1 μM or 3 μM to treat A375 Rac1 P29S or VRPP3 cells. PF 573228 was able to cancel the BRAFi resistance of both cell lines, supporting FAK as the critical target of defactinib, which can also inhibit PYK2.

Figure S13. Effects of avutometinib and VS-4718 on FAK and MAPK signaling proteins. Vector control A375 cells (VEC), cells expressing VAV1 or Rac1 P29S, and the spontaneously resistant VRPP3 subline harboring a Rac1 N92I mutant were treated with avutiometinib (VS-6766), FAK inhibitor VS-4718, or both drugs. Cell lysates were then immunoblotted for the indicated proteins.

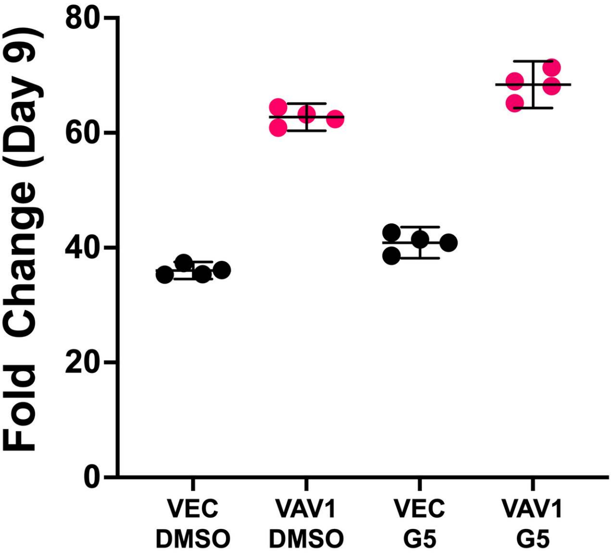

Figure S1

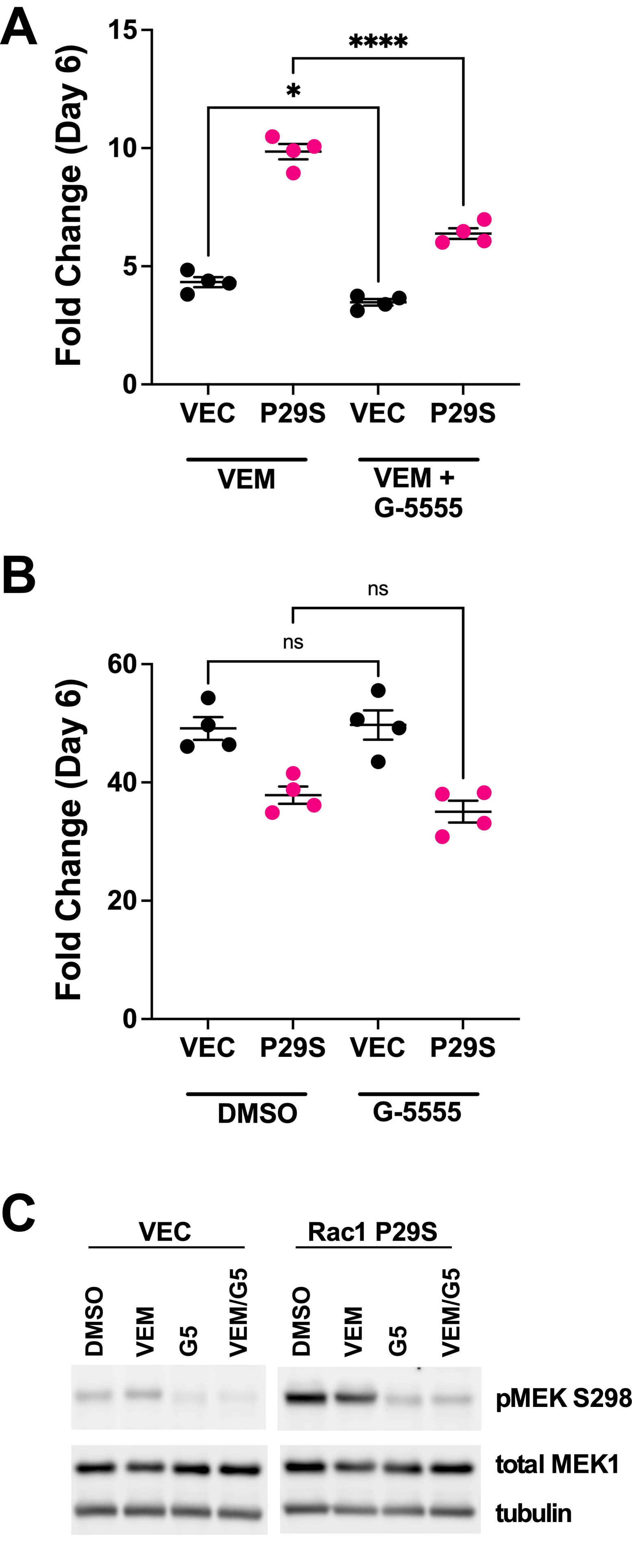
A
15
10
5
0
Fold Change (Day 6)
****
*
VEC
P29S
VEC
P29S
VEM
VEM +
G-5555
B
60
40
20
0
Fold Change (Day 6)
ns
ns
VEC
P29S
VEC
P29S
DMSO
G-5555
C
VEC
Rac1 P29S
DMSO
VEM
G5
VEM/G5
DMSO
VEM
G5
VEM/G5
pMEK S298
total MEK1
tubulin

Figure S2

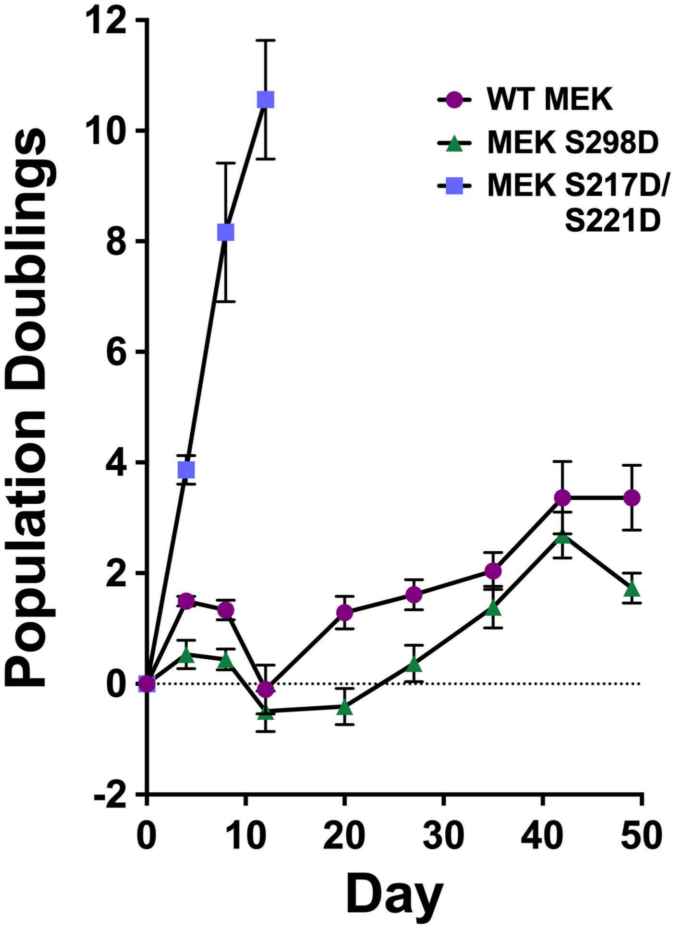

Population Doublings
12
10
8
6
4
2
0
-2
0
10
20
30
40
50
Day
WT MEK
MEK S298D
MEK S217D/
S221D

Figure S3

A

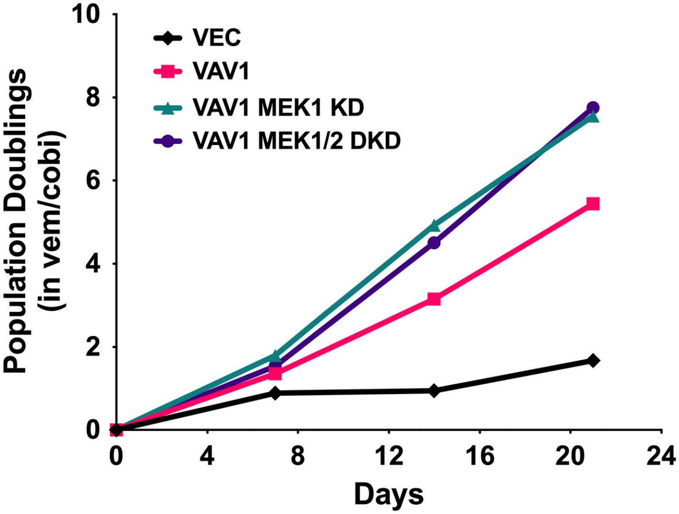

10
8
6
4
2
0
Population Doublings (in vem/cobi)
0
4
8
12
16
20
24
Days
VEC
VAV1
VAV1 MEK1 KD
VAV1 MEK1/2 DKD

B

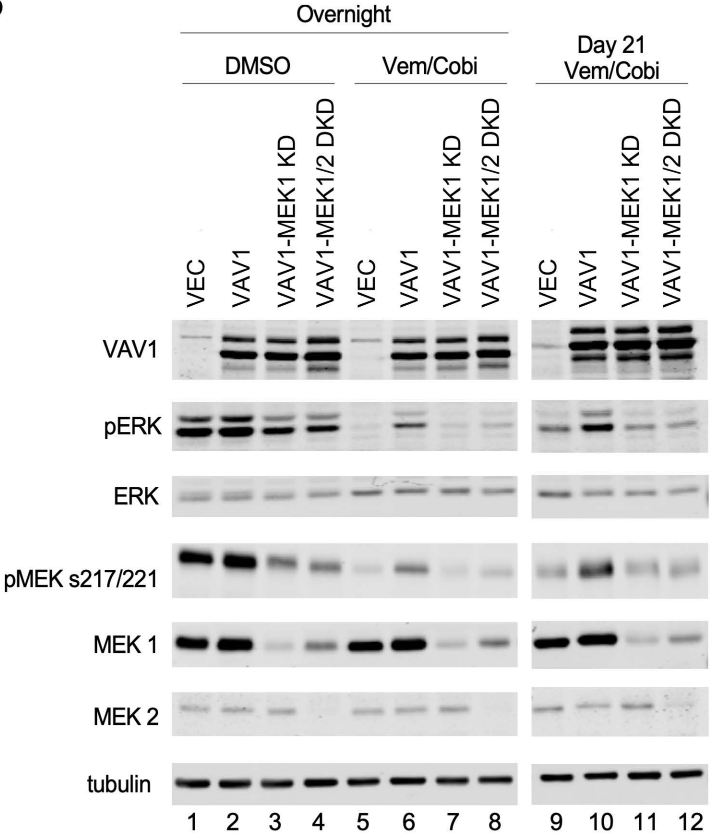

Overnight
DMSO
Vem/Cobi
Day 21 Vem/Cobi
VEC
VAV1
VAV1-MEK1 KD
VAV1-MEK1/2 DKD
VEC
VAV1
VAV1-MEK1 KD
VAV1-MEK1/2 DKD
VEC
VAV1
VAV1-MEK1 KD
VAV1-MEK1/2 DKD
VAV1
pERK
ERK
pMEK s217/221
MEK 1
MEK 2
tubulin
1
2
3
4
5
6
7
8
9
10
11
12

Figure S4

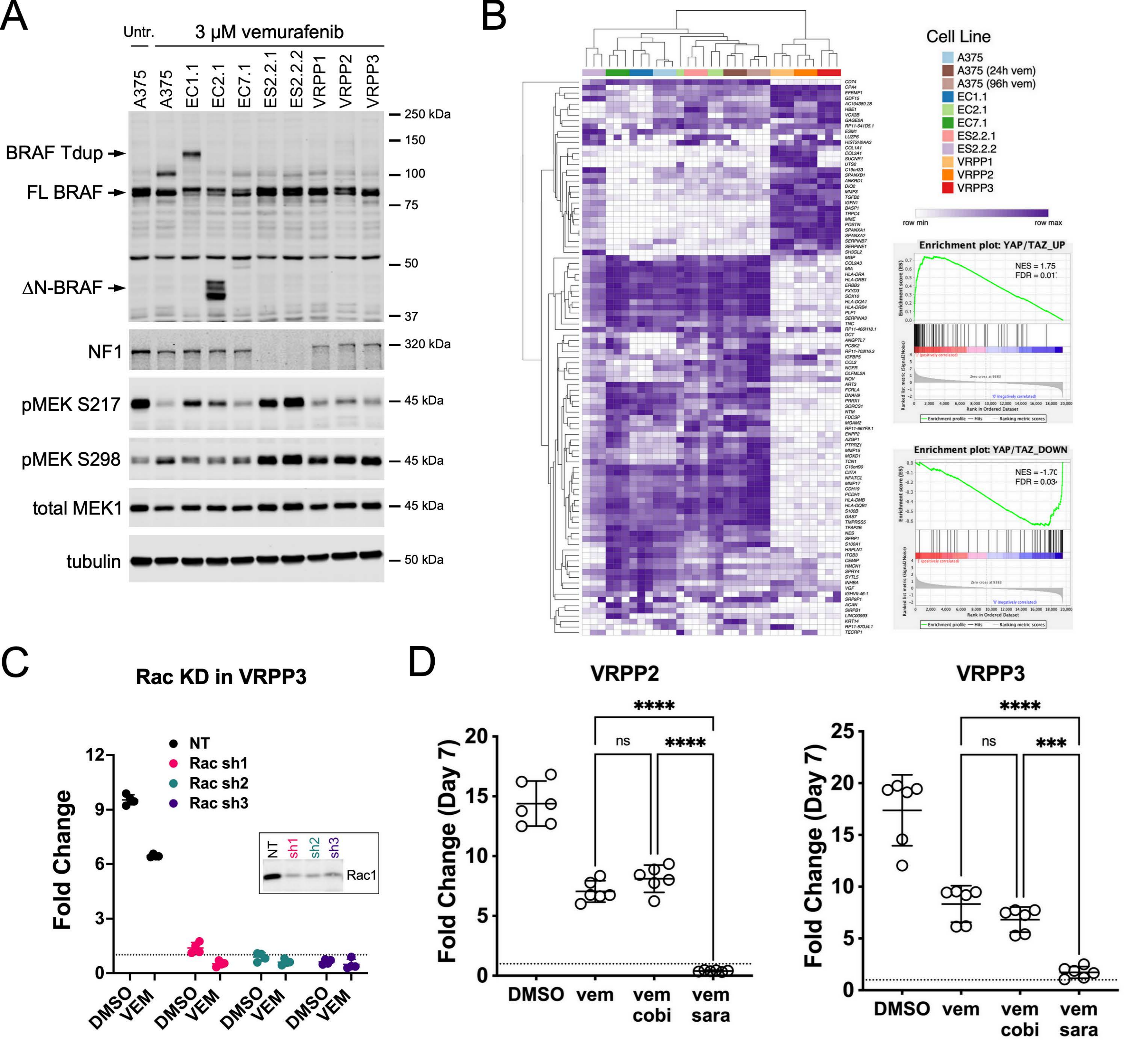
A
Untr.
3 μM vemurafenib
A375
A375
EC1.1
EC2.1
EC7.1
ES2.2.1
ES2.2.2
VRPP1
VRPP2
VRPP3
BRAF Tdup
FL BRAF
ΔN-BRAF
NF1
pMEK S217
pMEK S298
total MEK1
tubulin
250 kDa
150
100
75
50
37
320 kDa
45 kDa
45 kDa
45 kDa
50 kDa
B
Cell Line
A375
A375 (24h vem)
A375 (96h vem)
EC1.1
EC2.1
EC7.1
ES2.2.1
ES2.2.2
VRPP1
VRPP2
VRPP3
row min
row max
Enrichment plot: YAP/TAZ_UP
NES = 1.75
Enrichment plot: YAP/TAZ_DOWN
C
Rac KD in VRPP3
NT
Rac sh1
Rac sh2
Rac sh3
Fold Change
Rac1
DMSO
VEM
D
VRPP2
VRPP3
Fold Change (Day 7)
ns
****
***
DMSO
vem
vem cobi
vem sara
Figure S5

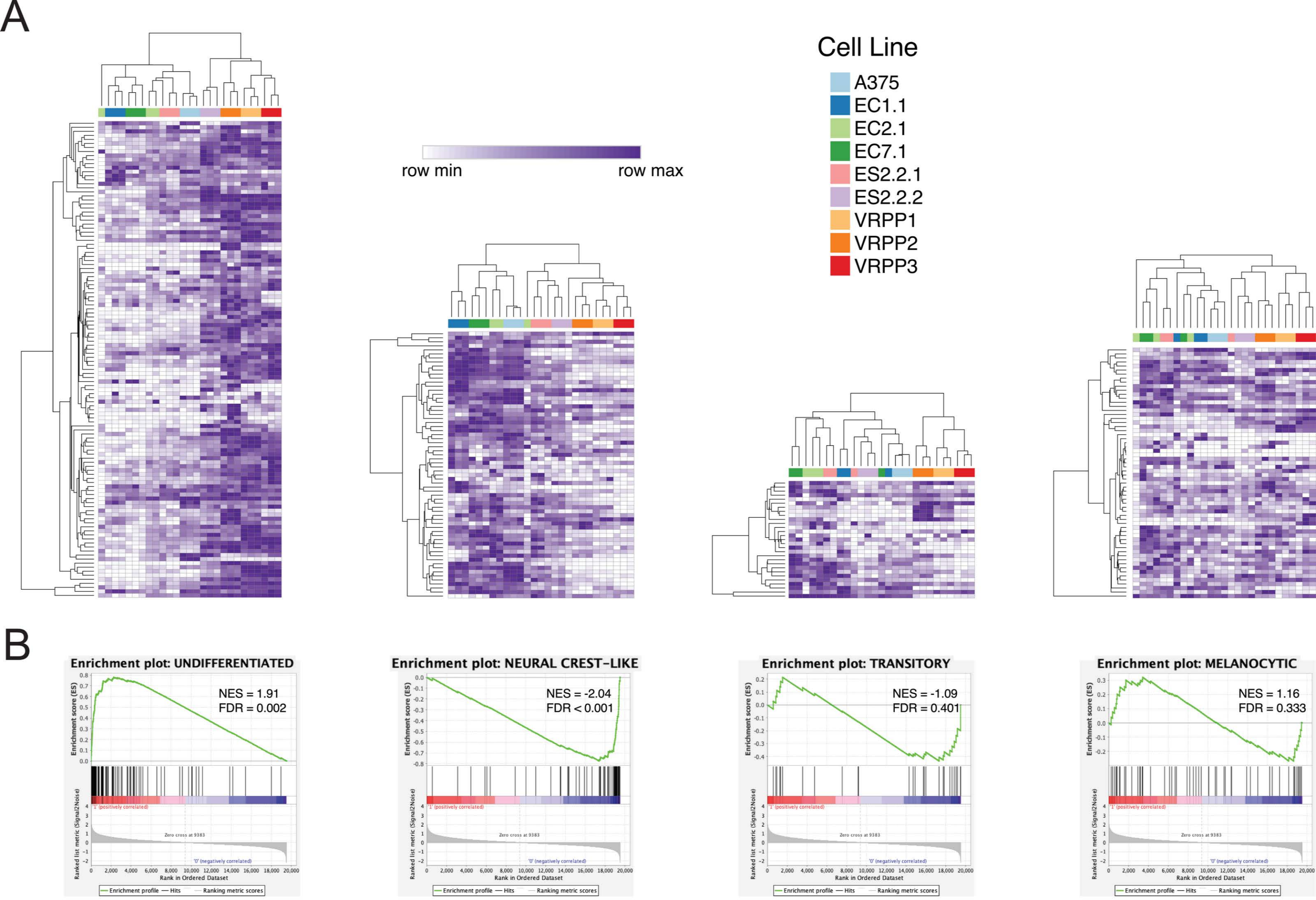
A
Cell Line
A375
EC1.1
EC2.1
EC7.1
ES2.2.1
ES2.2.2
VRPP1
VRPP2
VRPP3
row min
row max
B
Enrichment plot: UNDIFFERENTIATED
NES = 1.91
FDR = 0.002
Enrichment plot: NEURAL CREST-LIKE
NES = -2.04
FDR < 0.001
Enrichment plot: TRANSITORY
NES = -1.09
FDR = 0.401
Enrichment plot: MELANOCYTIC
NES = 1.16
FDR = 0.333
Enrichment score (ES)
Ranked list metric (Signal2Noise)
'1' (positively correlated)
Zero cross at 9383
'0' (negatively correlated)
Rank in Ordered Dataset
Enrichment profile
Hits
Ranking metric scores

Figure S6

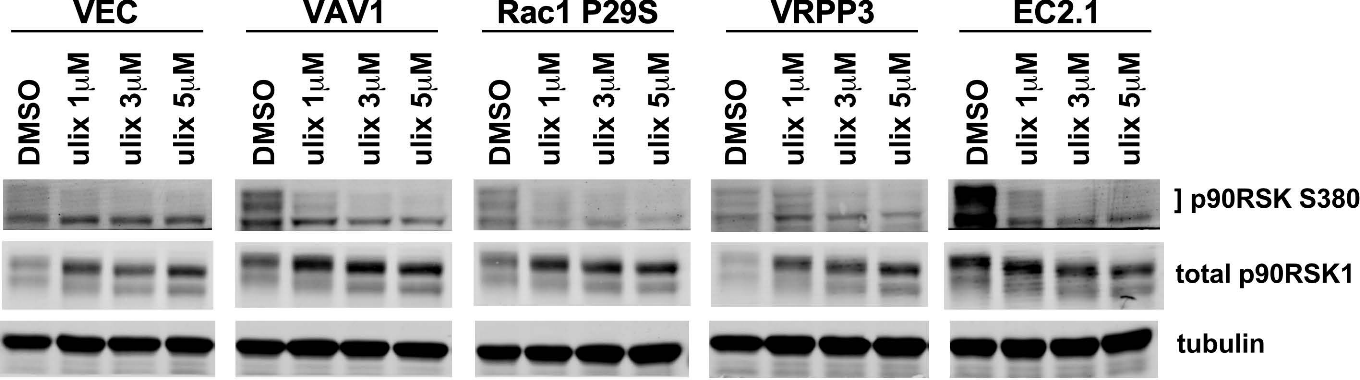
VEC
VAV1
Rac1 P29S
VRPP3
EC2.1
DMSO
ulix 1μM
ulix 3μM
ulix 5μM
] p90RSK S380
total p90RSK1
tubulin

Figure S7

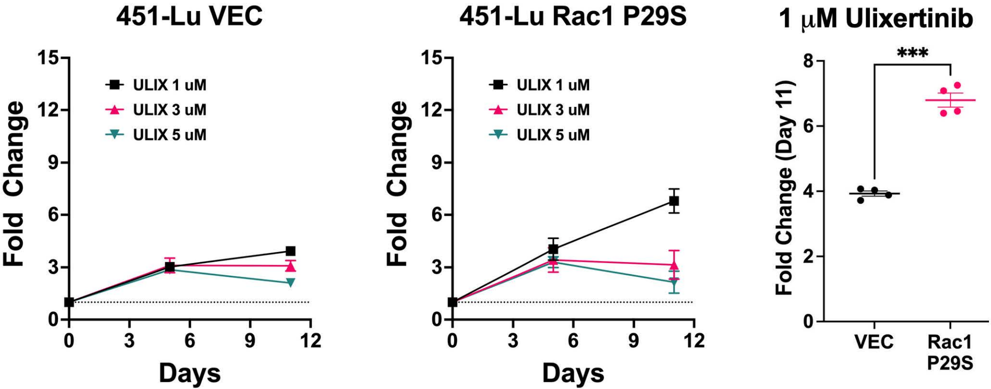
451-Lu VEC
ULIX 1 uM
ULIX 3 uM
ULIX 5 uM
Fold Change
Days
0
3
6
9
12
15
451-Lu Rac1 P29S
ULIX 1 uM
ULIX 3 uM
ULIX 5 uM
Fold Change
Days
1 μM Ulixertinib
***
Fold Change (Day 11)
0
2
4
6
8
VEC
Rac1 P29S

Figure S8

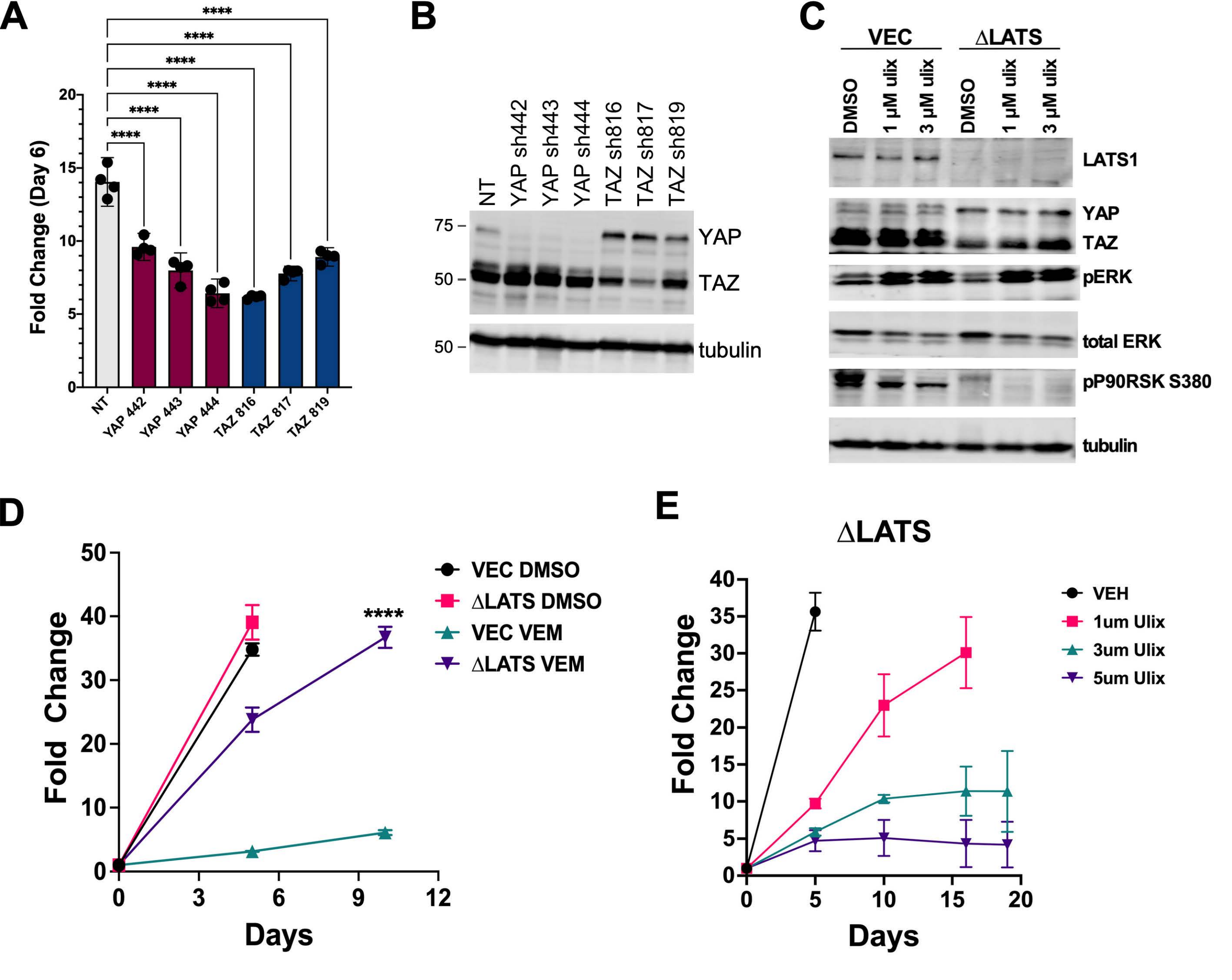
A
****
Fold Change (Day 6)
NT
YAP 442
YAP 443
YAP 444
TAZ 816
TAZ 817
TAZ 819
B
NT
YAP sh442
YAP sh443
YAP sh444
TAZ sh816
TAZ sh817
TAZ sh819
75
50
YAP
TAZ
tubulin
C
VEC
ΔLATS
DMSO
1 μM ulix
3 μM ulix
LATS1
YAP
TAZ
pERK
total ERK
pP90RSK S380
tubulin
D
Fold Change
Days
VEC DMSO
ΔLATS DMSO
VEC VEM
ΔLATS VEM
****
E
ΔLATS
Fold Change
Days
VEH
1um Ulix
3um Ulix
5um Ulix

Figure S9

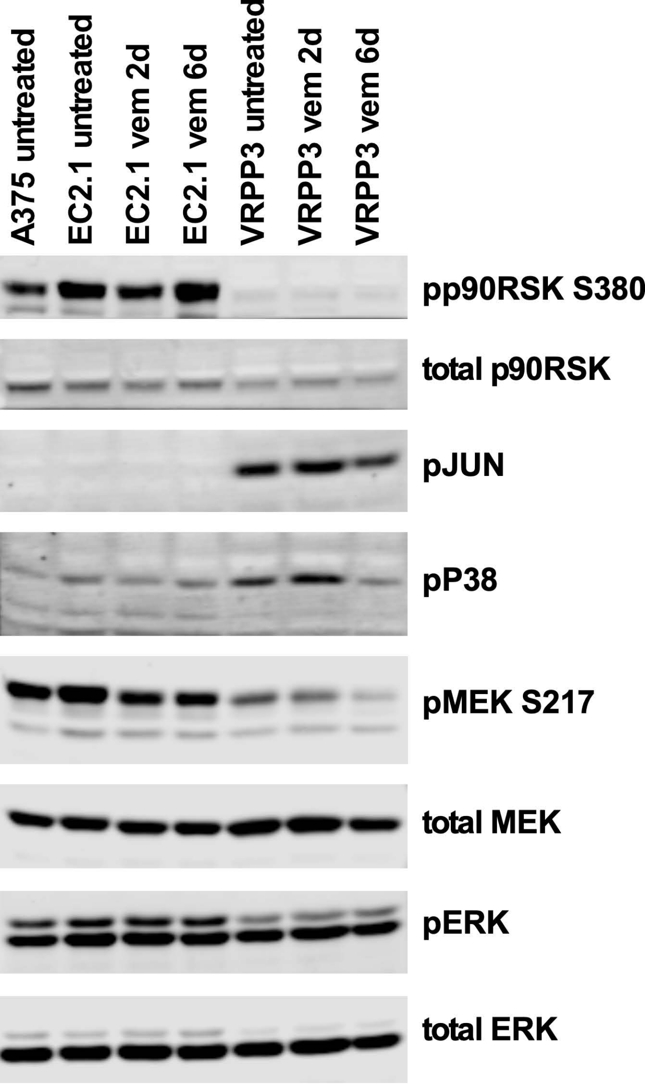
A375 untreated
EC2.1 untreated
EC2.1 vem 2d
EC2.1 vem 6d
VRPP3 untreated
VRPP3 vem 2d
VRPP3 vem 6d
pp90RSK S380
total p90RSK
pJUN
pP38
pMEK S217
total MEK
pERK
total ERK

Figure S10

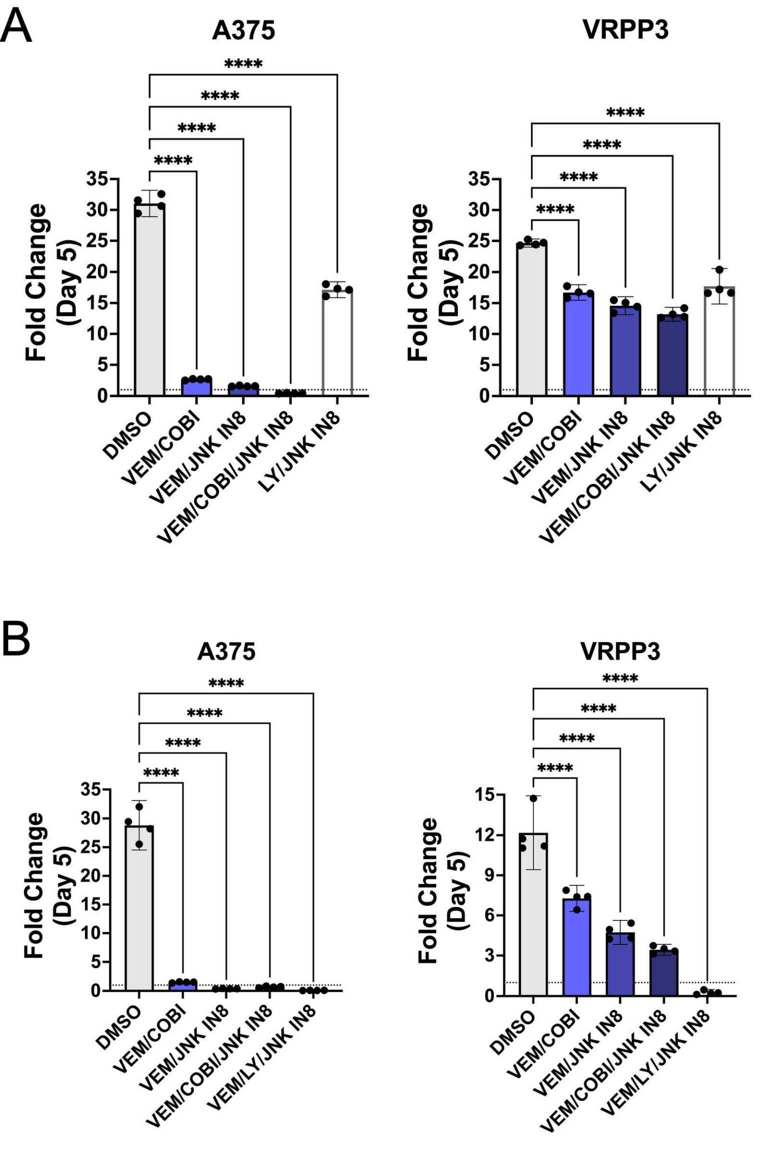
A
A375
VRPP3
****
Fold Change (Day 5)
35
30
25
20
15
10
5
0
DMSO
VEM/COBI
VEM/JNK IN8
VEM/COBI/JNK IN8
LY/JNK IN8
B
A375
VRPP3
12
9
6
3
VEM/LY/JNK IN8

Figure S11

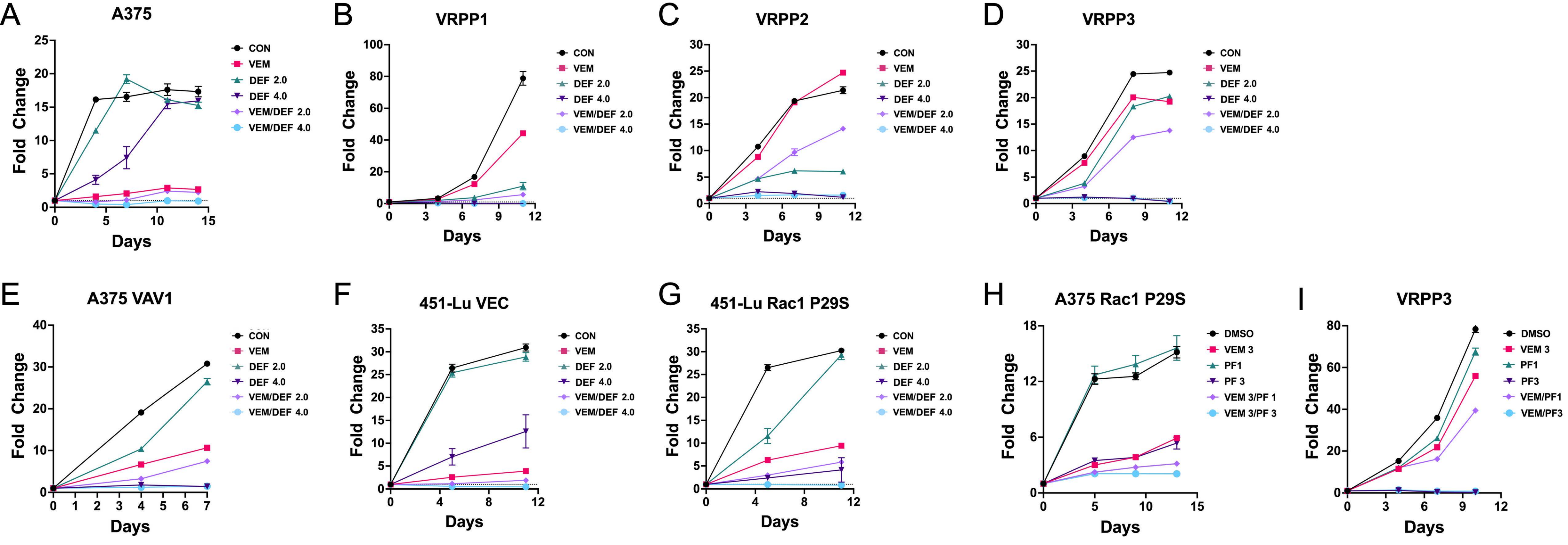
A
A375
B
VRPP1
C
VRPP2
D
VRPP3
E
A375 VAV1
F
451-Lu VEC
G
451-Lu Rac1 P29S
H
A375 Rac1 P29S
I
VRPP3
Fold Change
Days
CON
VEM
DEF 2.0
DEF 4.0
VEM/DEF 2.0
VEM/DEF 4.0
DMSO
VEM 3
PF1
PF 3
VEM 3/PF 1
VEM 3/PF 3
PF3
VEM/PF1
VEM/PF3

Figure S12

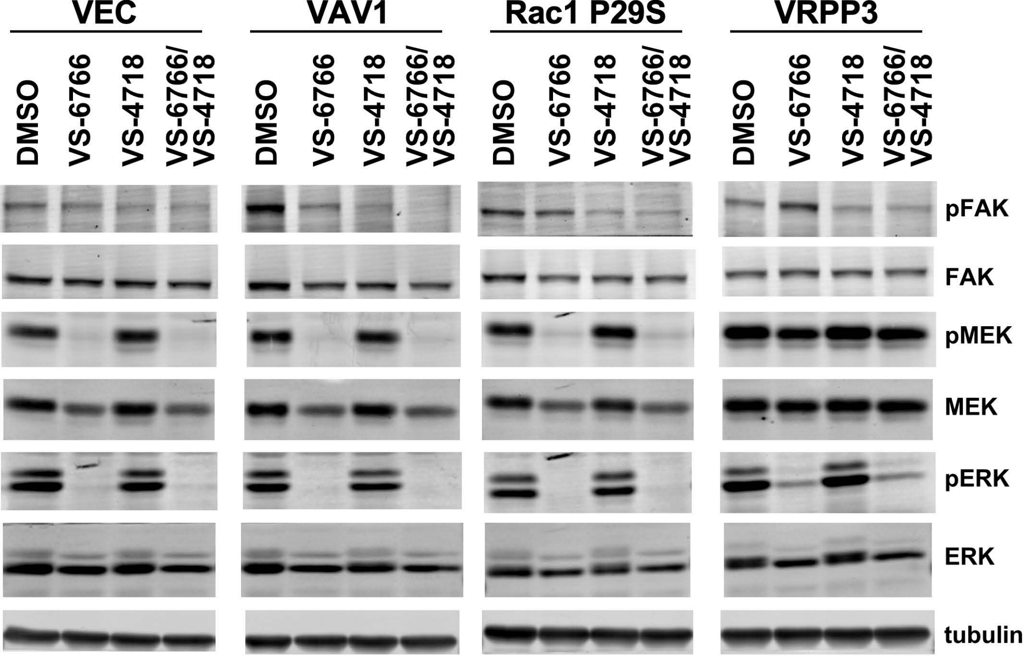
VEC
VAV1
Rac1 P29S
VRPP3
DMSO
VS-6766
VS-4718
VS-6766/ VS-4718
pFAK
FAK
pMEK
MEK
pERK
ERK
tubulin

Figure S13
