# Supplementary material for: A critical role of FAK signaling in Rac1-driven melanoma cell resistance to MAPK pathway inhibition
Source: Oncogene. 2025 Oct 18;44(46):4520–32. doi: 10.1038/s41388-025-03603-w (PMC12602353; doi:10.1038/s41388-025-03603-w)
Supplement: Supplementary file 4 — Supplemental Table S3 [file 41388_2025_3603_MOESM4_ESM.pdf]

Table S3. Pharmacological reagents, vectors, and antibodies used in this study

**Pharmacological reagents**		
**Compound**	**Target**	**Source**
Vemurafenib	BRAF V600E	MedChemExpress
G-5555	PAK1	Targetmol
Cobimetinib	MEK1/2	MedChemExpress
Ulixertinib	ERK1/2	Targetmol
Avutometinib (VS-6766)	MEK1/2	Targetmol (*in vitro* studies)<br>Verastem (*in vivo* studies)
Defactinib	FAK/Pyk2	Targetmol
VS-4718	FAK/Pyk2	Targetmol (*in vitro* studies)<br>Verastem (*in vivo* studies)
PF 573228	FAK	MedChemExpress
Saracatinib	SRC family kinases	MedChemExpress
JNK-IN-8	JNK	MedChemExpress
LY2228820	P38 MAPK	MedChemExpress

**Vectors**	
**Backbone**	**Insert**
PB-EF1α-MCS-PGK-G418	VAV1
PB-EF1α-MCS-PGK-G418	empty
PB-EF1α-MCS-PGK-Hygro	VAV1
PB-EF1α-MCS-PGK-Hygro	empty
PB-EF1α-MCS-PGK-Puro	Rac1 P29S
PB-EF1α-MCS-PGK-Puro	MEK1 wild type-myc
PB-EF1α-MCS-PGK-Puro	MEK1 S217D, S221D-myc
PB-EF1α-MCS-PGK-Puro	MEK1 S298D-myc
PB-EF1α-MCS-PGK-Puro	empty
pZIP-mCMV-ZsGreen-Puro	Rac1 sh1 (5'-CAAGGAGATTGGTGCTGTAAAA-3')
pZIP-mCMV-ZsGreen-Puro	Rac1 sh2 (5'-CCAAGAAGATTATGACAGATTA-3')
pZIP-mCMV-ZsGreen-Puro	Rac1 sh3 (5'-CGAATATATCCCTACTGTCTTA-3')
pZIP-mCMV-ZsGreen-Puro	YAP1 sh442 (5'-AGAAAGCTTTCTTACATGGTT-3')
pZIP-mCMV-ZsGreen-Puro	YAP1 sh443 (5'-CACATCGATCAGACAACAACA-3')
pZIP-mCMV-ZsGreen-Puro	YAP1 sh444 (5'-AGGTGATACTATCAACCAAAT-3')
pZIP-mCMV-ZsGreen-Puro	TAZ sh816 (5'-TCCGGAGGACTTCCTCAGCAA-3')
pZIP-mCMV-ZsGreen-Puro	TAZ sh817 (5'-CACATAGAAAAAATCACCACA-3')
pZIP-mCMV-ZsGreen-Puro	TAZ sh819 (5'-CCGGAGGACTTCCTCAGCAAT-3')
pZIP-mCMV-ZsGreen-Puro	Non-targeting
pSIREN-RetroQ-Puro	shMEK1 (5'-CCGCAGAGAGAGCAGATTTGA-3')
pSIREN-RetroQ-Hygro	shMEK2 (5'-CTCAAAGACGATGACTTCGAA-3')
pSIREN-RetroQ-Hygro	PAK1 sh3 (5'-GAGGATTACAATTCTTCTAAT-3')
pSIREN-RetroQ-Hygro	PAK2 sh3 (5'-CAGGAGGTTGCTATCAAACAA-3')
pSIREN-RetroQ-Puro	Non-targeting
pSIREN-RetroQ-Hygro	Non-targeting
plentiCRISPRv2	LATS1 sgRNA (5'-GGTATCCAAGAAGGGTGTGT-3')
plentiCRISPRv2	LATS2 sgRNA (5'-GAGTGTCCACCTTACAAGCA-3')

Antibodies			
**Antibody**	**Clone**	**Catalog No.**	**Source**
Rabbit anti-BRAF V600E	RM8	MA5-24661	Thermo Fisher Scientific
Mouse anti-ERK1/2	L3F12	4696	Cell Signaling Technology
Rabbit anti-p-ERK1/2 T202/Y204	D13.14.4E	4370	Cell Signaling Technology
Rabbit anti-LATS1	C66B5	3477	Cell Signaling Technology
Mouse anti-MEK1	61B12	2352	Cell Signaling Technology
Rabbit anti-MEK2	polyclonal	9125	Cell Signaling Technology
Rabbit anti-p-MEK S298	D1P9E	98195	Cell Signaling Technology
Rabbit anti-p-MEK S217/221	41G9	9154	Cell Signaling Technology
Rabbit anti-NF1	D7R7D	14623	Cell Signaling Technology
Rabbit anti-p90RSK	32D7	9355	Cell Signaling Technology
Rabbit anti-p-p90RSK S380	D3H11	11989	Cell Signaling Technology
Rabbit anti-PAK1	Polyclonal	2602	Cell Signaling Technology
Rabbit anti-PAK2	C17A10	2615	Cell Signaling Technology
Rabbit anti-p-c-JUN S73	D47G9	3270	Cell Signaling Technology
Rabbit anti-p-P38 T180/Y182	D3F9	4511	Cell Signaling Technology
Mouse anti-Rac1	102	610651	BD Biosciences
Mouse anti-tubulin	12G10		DSHB
Rabbit anti-Vav1	polyclonal	HPA001864	Sigma
Rabbit anti-YAP/TAZ	D24E4	8418	Cell Signaling Technology
